# Functionality of primary hepatic non-parenchymal cells in a 3D spheroid model and contribution to acetaminophen hepatotoxicity

**DOI:** 10.1007/s00204-020-02682-w

**Published:** 2020-02-28

**Authors:** Catherine C. Bell, Bhavik Chouhan, Linda C. Andersson, Håkan Andersson, James W. Dear, Dominic P. Williams, Magnus Söderberg

**Affiliations:** 1 CVRM Safety, Clinical Pharmacology and Safety Sciences, R&D, AstraZeneca, Gothenburg, Sweden; 2 Functional and Mechanistic Safety, Clinical Pharmacology and Safety Sciences, R&D, AstraZeneca, Gothenburg, Sweden; 3 Clinical Pharmacology and Quantitative Pharmacology, Clinical Pharmacology and Safety Sciences, R&D, AstraZeneca, Gothenburg, Sweden; 4 Centre for Cardiovascular Science, The Queen’s Medical Research Institute, University of Edinburgh, Edinburgh, UK; 5 Functional and Mechanistic Safety, Clinical Pharmacology and Safety Sciences, R&D, AstraZeneca, Cambridge, UK

**Keywords:** Hepatotoxicity, 3D models, miRNA, In vitro toxicology

## Abstract

In addition to hepatocytes, the liver comprises a host of specialised non-parenchymal cells which are important to consider in the development of in vitro models which are both physiologically and toxicologically relevant. We have characterized a 3D co-culture system comprising primary human hepatocytes (PHH) and non-parenchymal cells (NPC) and applied it to the investigation of acetaminophen-induced toxicity. Firstly, we titrated ratios of PHH:NPC and confirmed the presence of functional NPCs via both immunohistochemistry and activation with both LPS and TGF-β. Based on these data we selected a ratio of 2:1 PHH:NPC for further studies. We observed that spheroids supplemented with NPCs were protected against acetaminophen (APAP) toxicity as determined by ATP (up to threefold difference in EC_50_ at day 14 compared to hepatocytes alone) and glutathione depletion, as well as miR-122 release. APAP metabolism was also altered in the presence of NPCs, with significantly lower levels of APAP-GSH detected. Expression of several CYP450 enzymes involved in the bioactivation of APAP was also lower in NPC-containing spheroids. Spheroids containing NPCs also expressed higher levels of miRNAs which have been implicated in APAP-induced hepatotoxicity, including miR-382 and miR-155 which have potential roles in liver regeneration and inflammation, respectively. These data indicate that the interaction between hepatocytes and NPCs can have significant metabolic and toxicological consequences important for the correct elucidation of hepatic safety mechanisms.

**Electronic supplementary material:**

The online version of this article (10.1007/s00204-020-02682-w) contains supplementary material, which is available to authorized users.

## Introduction

The liver is a key organ in the detoxification of chemicals and drugs, performing a multitude of metabolic reactions which ultimately result in clearance and excretion. It is also a frequent site of drug-induced toxicity reactions, with hepatotoxicity a leading cause of post-marketing drug withdrawals (Onakpoya et al. 2016). This is, in part, due to its exposure to chemically reactive metabolites formed as a result of bioactivation (Park et al. 2011). Predicting the potential of a new medicine to cause drug-induced liver injury (DILI) in man is, however, complicated by species differences in both the expression and activity of a number of proteins involved in the absorption, distribution, metabolism, and excretion (ADME) of drugs (Martignoni et al. 2006). In vitro systems which incorporate well-characterised human cells and/or tissue are therefore becoming increasingly important in the development of safe medicines (Baker et al. 2018; Godoy et al. 2013; Weaver and Valentin 2019).

Though hepatocytes are the predominant cell of the liver, comprising approximately 80% of its total mass, a number of other, specialised cells with key functions are also present. These include Kupffer cells, hepatic resident macrophages, stellate cells which produce collagen and store vitamin A, and liver sinusoidal endothelial cells (LSECs), which provide a permeable barrier between the blood and the space of Disse. In addition to their individual functions, the communication between these cells through the release of soluble factors such as cytokines and chemokines is key to liver homeostasis, including the response to invading pathogens, or toxic insult (Robinson et al. 2016). In the case of DILI, non-parenchymal cells may be directly targeted or activated in response to the release of damage-associated molecular patterns (DAMPs), such as intracellular proteins or nucleotides from damaged hepatocytes (Godoy et al. 2013).

Despite a drive for more patient-relevant assays to be included at an earlier stage in drug development, modelling hepatotoxicity in vitro can be a difficult task. Although researchers have access to primary human cells, the loss of phenotype that occurs upon plating in 2D is a huge barrier in the investigation of DILI, particularly where metabolic activation is required (Heslop et al. 2016; Lauschke et al. 2016a, b; Rowe et al. 2010). Three-dimensional hepatocyte spheroids have recently emerged as a promising in vitro model in which the phenotype of primary cells can be maintained for several weeks (Bell et al. 2016). Although the possibility of including additional non-parenchymal cells (NPCs) such as Kupffer cells (Messner et al. 2013) stellate cells and liver sinusoidal endothelial cells (Bell et al. 2016; Proctor et al. 2017) has been investigated, little has been reported surrounding their role in drug toxicity. Furthermore, little is known about the behaviour of NPCs when cultured in more advanced in vitro models both in terms of their phenotype and function.

In addition to considering the cell types included in hepatic model systems, the choice of relevant endpoints which are applicable to human toxicity is also extremely important. Measuring biomarkers of liver injury which can also be monitored in patient populations increases the translatability of any in vitro assay. In particular, miRNAs are of significant interest due to their relative stability in biological fluids, frequent tissue specificity and conservation between different species. Indeed, miR-122 represents one of the few biomarker candidates that can be monitored across preclinical animal models, in vitro cell assays and in the clinic (Kia et al. 2015a, b; Lewis et al. 2011; Wang et al. 2009). It is highly liver-specific and may be more sensitive than traditional protein biomarkers in identifying patients at risk of developing severe liver injury (Antoine et al. 2013).

In the current study, we have generated a co-culture model containing both primary human hepatocytes (PHH) and NPC, and used this model to investigate how NPCs influence the cytotoxicity of the model hepatotoxic compound acetaminophen (APAP).

## Methods

### Cell culture media

Maintenance media comprised Williams E media supplemented with 2 mM L-glutamine, 100 U/ml penicillin, 100 μg/ml streptomycin, 10 μg/ml insulin, 5.5 μg/ml transferrin, 6.7 ng/ml sodium selenite and 100 nM dexamethasone.

### Generation of mono- and co-culture spheroids

Cryopreserved primary cells from a single hepatocyte donor and four NPC donors were purchased from BioIVT and Lonza, respectively. Characteristics of these donors are reported in Table 1.

**Table 1:** Characteristics of cryopreserved hepatocyte and non-parenchymal cell donors

Cells	Sex	Age	Race	Cause of death
PHH1	Female	78	Caucasian	Stroke
NPC1	Female	43	Hispanic	Unknown
NPC2	Male	12	Caucasian	Unknown
NPC3	Female	55	Caucasian	Unknown
NPC4	Female	19 months	African American	Unknown

*PHH *primary human hepatocyte, *NPC *non-parenchymal cell

Cryopreserved hepatocytes were thawed according to the suppliers’ instructions, resuspended in maintenance medium supplemented with 10% foetal bovine serum (FBS; Gibco) and counted. 1500 cells/well were then seeded in ultra-low attachment plates (Corning) and centrifuged for 2 min at 125 × *g*. For co-culture spheroids, mixed NPCs were added to the cell suspension at various ratios prior to centrifugation. All spheroids contained 1500 hepatocytes. After 4–5 days a 50% media change was performed with FBS-free media and this was repeated daily until day 8 after seeding, when treatments began and which will now be referred to as day 0.

### NPC activation with LPS and/or TGF-β

At day 0, day 7, or day 14 spheroids were challenged with lipopolysaccharide (LPS; 10 μg/ml; Sigma) for 48 h, after which media was collected and stored at −80 ℃ until analysis. The amount of secreted IL-6 was then quantified via high sensitivity ELISA (Invitrogen), according to the manufacturer’s instructions. To assess stellate cell activation, spheroids were challenged with 10 ng/ml TGF-β (Sigma) at day 0 and collected for immunohistochemistry and mRNA analysis after 72 h.

### APAP treatment and viability and glutathione measurements

Acetaminophen (0–10 mM) was diluted in maintenance media and a full media change with the compound was performed every 2–3 days. At each timepoint cellular ATP content was determined using the Cell-TiterGlo reagents (Promega). Media was removed and 25 µl of the reagent was added. After a 20 min incubation at 37 ℃, luminescence was measured. Total glutathione was determined using the GSH-glo kit (Promega) according to the manufacturers instructions. All measurements were obtained from individual spheroids. Dose–response curves were generated in GraphPad Prism 8.0.1.

### miRNA and mRNA analysis

Total RNA was isolated from spheroids or media samples (50 µl) using the miRNeasy kit from Qiagen.

For mRNA analysis, approximately 20 spheroids were pooled and 75 ng RNA was reverse transcribed using the High Capacity cDNA reverse transcription kit (Applied Biosystems). TaqMan primers and probes for each gene of interest were purchased from Applied Biosystems. Data were analysed using the ΔΔCt method against the reference gene GAPDH.

For miRNA analysis, six spheroids were collected. cDNA was prepared from 2 µl total RNA using the TaqMan Advanced miRNA cDNA synthesis kit (Applied Biosystems) and expression of a panel of miRNAs was assessed by qPCR using TaqMan miRNA advanced probes. Relative expression of each miRNA was normalised to the endogenous U6 snRNA using the ΔΔCt method. For media samples, a standard curve was generated using synthetic miR-122 and unknown samples were interpolated accordingly.

### APAP metabolism

50 µl media from APAP-treated spheroids was collected and quenched with 150 µl acetonitrile containing 15 nM of the internal standard (IS) 5,5-diethyl-1,3-diphenyl-2-iminobarbituric acid and 0.8% formic acid. Samples were centrifuged (2100 × *g*, 20 min, 4 °C) and 50 µl of the resulting supernatant was diluted 1:3 with ddH_2_O and further dilutions were performed with acetonitrile:ddH_2_O (19:81 v/v) containing 0.15% formic acid and 2.8 nM IS. Quantification was achieved on a triple-quadrupole mass spectrometer (XevoTQ-S; Waters, Milford, MA, USA) equipped with an Acquity ultraperformance liquid chromatography (UPLC) I-Class system (Waters Corporation, MA, USA). The MS system was equipped with an electrospray ionisation source and settings were as follows: capillary voltage 0.5 kV; desolvation temperature 600 °C; cone gas flow 150 l/h; nebulizer gas 7.0 bar; collision gas flow 0.15 ml/min. MSMS optimization was performed for all the metabolites and conditions are specified in Table 2. Separation was performed using a Waters Acquity UPLC^®^ HSS T3 column (50 mm × 2.1 mm, 1.8 μm) fitted with a column heater set to 40 °C. The mobile phase consisted of solvent A (0.1% formic acid in dd-H_2_O) and solvent B (0.1% formic acid in acetonitrile). The elution profile was: 0.2% B, 0.00 to 0.30 min; linear gradient to 15% B, 0.31 to 1.80 min; isocratic hold, 1.81 to 2.40 min; 5% B, 2.41 to 2.46 min; re-equilibration to 0.2% B 2.47 to 3 min. The flow rate was 1.0 ml/min. The injection volume was 2 μl.

**Table 2:** MS parameters for determination of APAP and metabolites

Metabolite	MRM (parent → daughter) m/z	Dwell (s)	Cone voltage (V)	Collision energy (V)
APAP	152.01 → 109.96	0.014	40	16
APAP-glucuronide	328.2 → 152.15	0.014	15	20
APAP-sulfate	229.93 → 149.89	0.014	10	22
APAP-GSH	457.16 → 139.97	0.014	50	40
5,5-Diethyl-1,3-diphenyl-2-iminobarbituric acid (IS)	336.21 → 195.00	0.014	40	16

### Immunohistochemistry

Spheroids were collected and pooled, washed in PBS, and fixed in formalin for 1 h at room temperature. After washing twice in PBS, spheroids were transferred to a cryomold and suspended in Histogel, before paraffin embedding and sectioning (4 μm). Cellular morphology was assessed via hematoxylin and eosin (H+E) staining, and immunohistochemistry was performed using the Ventana Ultra and associated reagents from Roche. Details of the conditions for each of the antibodies used are reported in Table 3.

**Table 3:** Summary of antibodies and conditions used for immunohistochemistry on the Ventana Ultra

Antibody	Supplier	Host	Pretreatment	Dilution
CD68	Novocastra	Mouse	mCC1, pH8	1:100
α-SMA	Dako	Mouse	mCC1, pH8	1:100
COL1A1	Biosite	Rabbit	stdCC1, pH8	1:1000

## Results

### Characterisation of NPC-containing spheroids

**Fig. 1:**
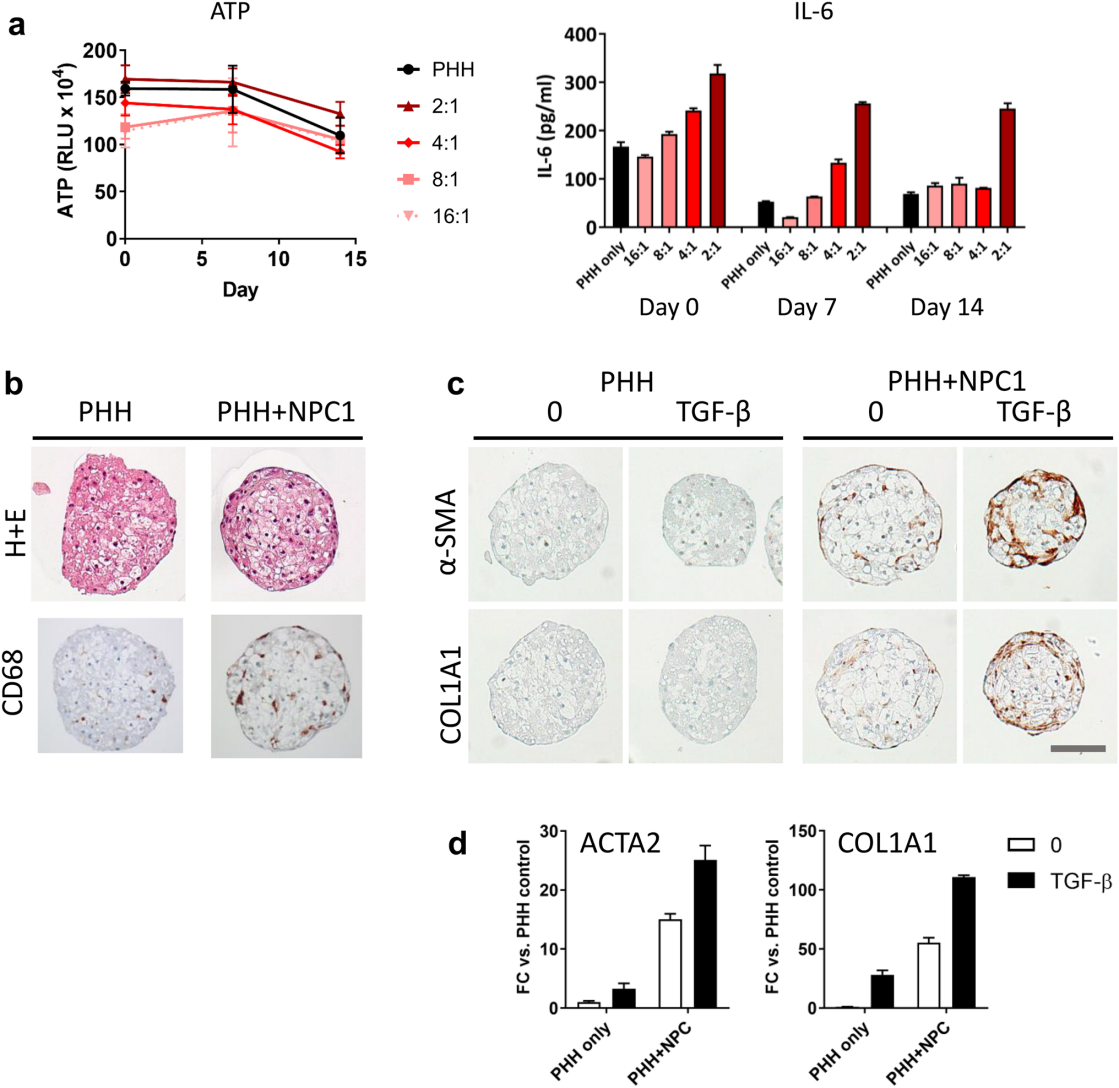
Characterisation of primary human hepatocyte spheroids supplemented with non-parenchymal cells. **a** The number of supplementary non-parenchymal cells was titrated and viability was assessed at different timepoints by measuring cellular ATP content. Data represent the average of six spheroids ± SD. The black line indicates hepatocyte only spheroids. All spheroids contained 1500 hepatocytes. **b** LPS-induced secretion of IL-6 was measured periodically to determine Kupffer cell function. Media from six spheroids was pooled after 48 h stimulation with 10 µg/ml LPS. In the absence of LPS, < 15 pg/ml IL-6 was detected from all conditions. **c** Histological assessment of 3D spheroids via H + E staining and immunohistochemistry for markers of Kupffer cells (CD68) indicates the presence NPCs. Scale bar represents 100 µm. **d** Increased expression of activated stellate cell markers α-SMA and COL1A1 following activation of hepatic stellate cells with TGF-β (10 ng/ml). Spheroids were pooled after 48 h and either lysed with Qiazol for RNA isolation or fixed with formalin for IHC

To determine the optimal number of NPCs for co-culture with primary human hepatocytes, the ratio of PHH:NPC was titrated. All spheroids contained 1500 hepatocytes, but NPCs were added at the indicated ratios. Addition of supplementary NPCs did not have any detrimental effect on spheroid viability, with equivalent ATP levels measured in all conditions (Fig. 1a). The presence of functional Kupffer cells was confirmed through the measurement of secreted IL-6 following LPS challenge. Although hepatocyte only spheroids also responded to LPS treatment, increased IL-6 was detected with the addition of increasing numbers of NPCs (Fig. 1b). After 14 days in culture only the spheroids with the greatest number of NPCs (2:1) were still responsive to LPS challenge, likely due to either a loss of the cells from the spheroid or a loss of functionality. Based on these data the 2:1 ratio was selected for further experiments. Cellular morphology was assessed via H+E staining and established that viable cells were present at the core of the spheroid (Fig. 1b). The presence of Kupffer cells (CD68 +) was confirmed using immunohistochemistry, which showed an even distribution of the NPCs throughout the spheroid (Fig. 1b). Stellate cells exist in multiple activation states which can be difficult to track, particularly when they are cultured in vitro. Here, we observed faint α-SMA staining, a classical marker of activated stellate cells, in untreated spheroids supplemented with NPCs. To understand whether this represented true activation of the stellate cells, spheroids were treated with TGF-β, whereupon a significant increase in α-SMA and COL1A1 was observed at both the protein and RNA level (Fig. 1c, d). This suggests that the cells are not fully activated merely by the culture conditions and that they can be pushed towards a more activated state with the addition of classical inducers. The biliary epithelial cell marker cytokeratin 19 was not detected in these spheroids (Supplemetary Fig. 1a).

### NPCs protect against APAP-induced toxicity

**Fig. 2:**
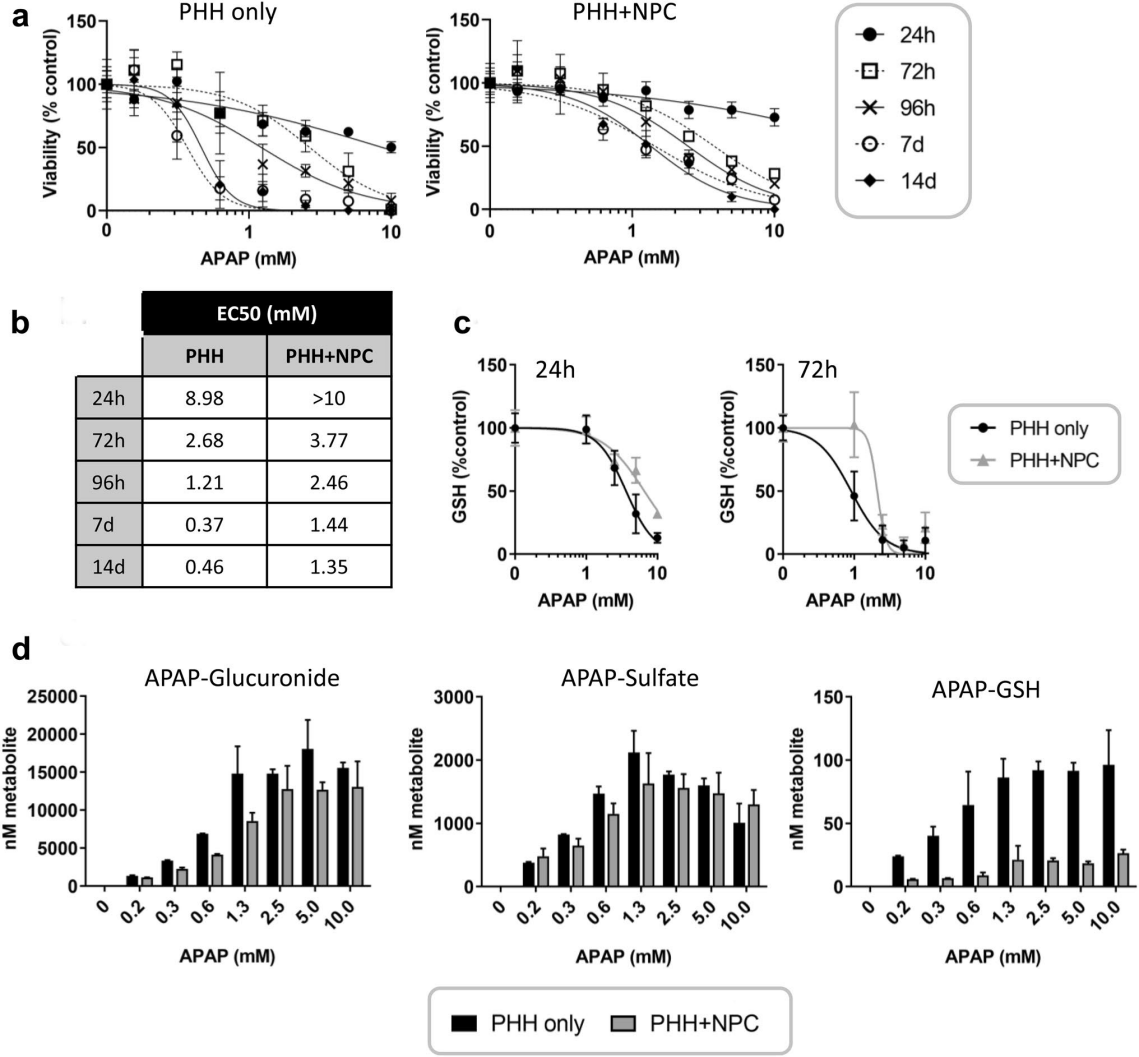
Hepatic non-parenchymal cells protect against acetaminophen toxicity. **a** Cytotoxicity of APAP was determined by measuring cellular ATP at various timepoints. PHH only spheroids (left panel) were more sensitive to APAP toxicity than spheroids containing NPCs (right panel), particularly after repeated treatments. **b** Concentrations resulting in a 50% reduction in viability (EC_50_) were calculated for both models—lower concentrations of APAP were required to cause a loss of viability in PHH only spheroids. **c** Glutathione depletion was also observed at lower concentrations of APAP when PHH were cultured in the absence of NPCs. This occurred prior to ATP depletion. Data represent the average ± SD of six spheroids. **d** APAP metabolites were measured in the media after 72 h treatment. Data represent the average ± SD of two biological replicates consisting of pooled media from three spheroids each

To assess how the addition of NPCs would impact upon drug-induced toxicity, spheroids were treated with the model hepatotoxicant acetaminophen (APAP) and the response was assessed via a panel of readouts. NPCs protected against APAP-induced toxicity, with higher concentrations required to cause a depletion in cellular ATP (Fig. 2a, b). A slight difference in sensitivity was seen already after 24/72 h treatment (Fig. 2b), and after repeated treatments of up to 14 days this difference increased to approximately threefold. At the same time, cellular glutathione was depleted to a greater extent in hepatocyte only spheroids, which might suggest they are more sensitive to APAP-induced oxidative stress (Fig. 2c). Glutathione depletion was an early event, occurring after 6 h APAP exposure (Supplementary Fig. 1b).

**Fig. 3:**
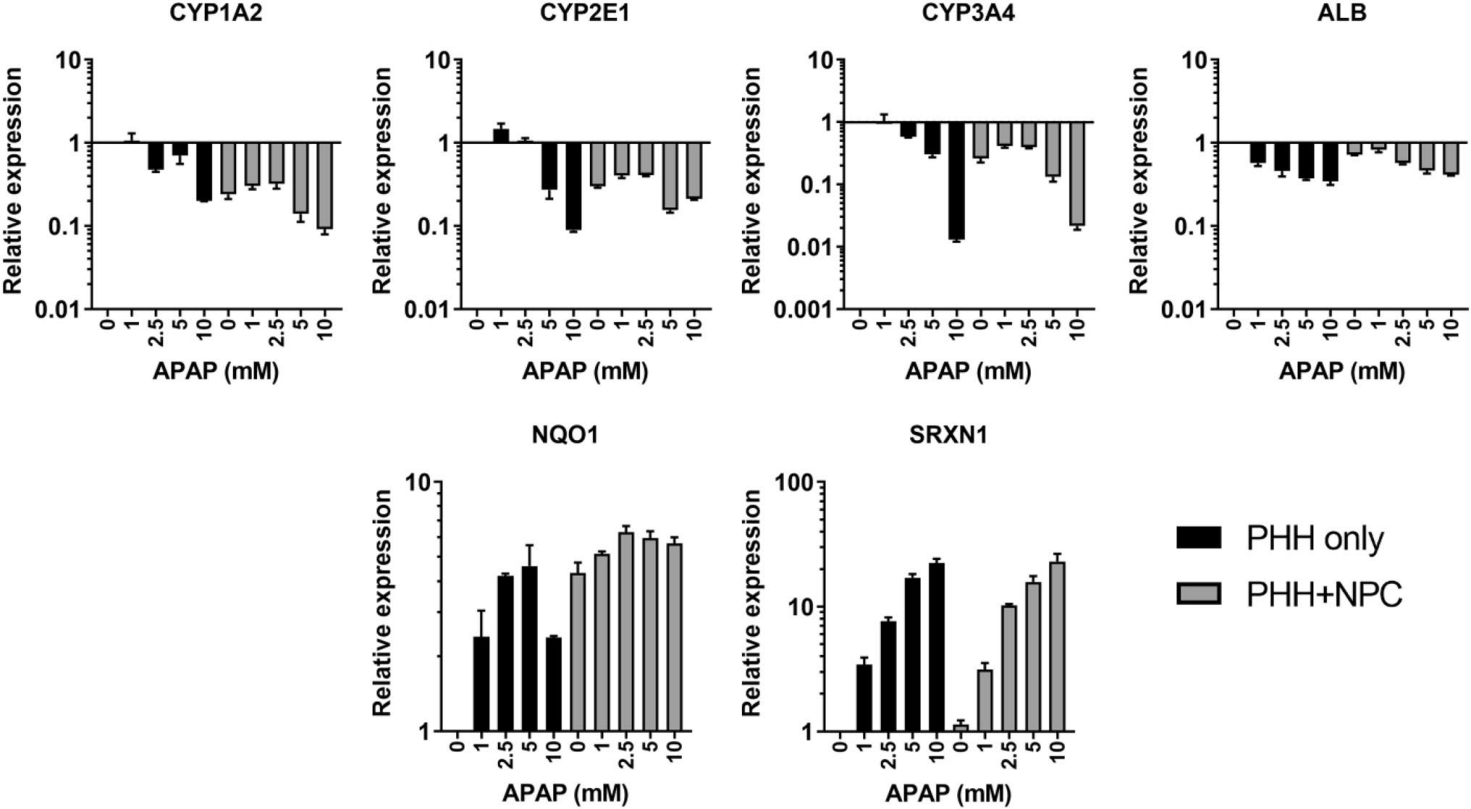
Acetaminophen induces changes in cytochrome P450 enzyme expression and markers of oxidative stress. CYP1A2, CYP2E1, and CYP3A4 have been implicated in the metabolism of APAP to the reactive metabolite NAPQI. Baseline expression of these enzymes was higher in PHH only spheroids (black bars) compared to PHH + NPC spheroids (grey bars). Albumin expression was assessed as a marker for hepatocytes. Data represent the average of three replicates, generated from RNA isolated from 24 pooled spheroids. GAPDH was used as a housekeeping gene and all data are relative to the untreated PHH only spheroids. Srxn1 and Nqo1 were investigated as markers of oxidative stress

The hepatotoxicity of APAP is thought to be related to the formation of the reactive metabolite, NAPQI, which is highly protein-reactive (McGill et al. 2013). We therefore assessed the formation of APAP metabolites in each cell model via LC–MS/MS. Although similar amounts of both the glucuronide and sulfate metabolite were measured in mono- and co-culture (Fig. 2d), less of the APAP-GSH conjugate (which may serve as a surrogate for NAPQI production) could be detected in media from NPC-containing spheroids. Both hepatocyte only and NPC-supplemented spheroids contained the same number of hepatocytes (1500 cells/spheroid). To understand whether this difference was related to differential expression of the enzymes involved in APAP metabolism, mRNA levels of CYP1A2, 2E1 and 3A4 were investigated (Fig. 3). Expression of each of these enzymes was lower in NPC-containing spheroids, despite equivalent albumin expression (CYP1A2 = − 4.4-fold; CYP2E1 = − 3.3-fold; CYP3A4 = − 3.8-fold; ALB = − 1.4-fold). In addition, APAP treatment resulted in a downregulation of each of these enzymes. This effect was more pronounced in PHH spheroids, particularly when considering that higher levels of each of CYPs were present initially. Expression of the Nrf-2 target genes Nqo1 and Srxn1 was also investigated and both were upregulated by APAP treatment, suggesting that oxidative stress was induced in both models. Basal expression of Nqo1 was higher in the NPC-containing spheroids and not as inducible as Srxn1. Co-incubation with the non-selective CYP inhibitor 1-aminobenzotriazole (1 mM) resulted in a modest protective effect (Supplemetary Fig. 1c).

### Differential expression and release of hepatic miRNAs in hepatocyte only and NPC-containing spheroids

**Fig. 4:**
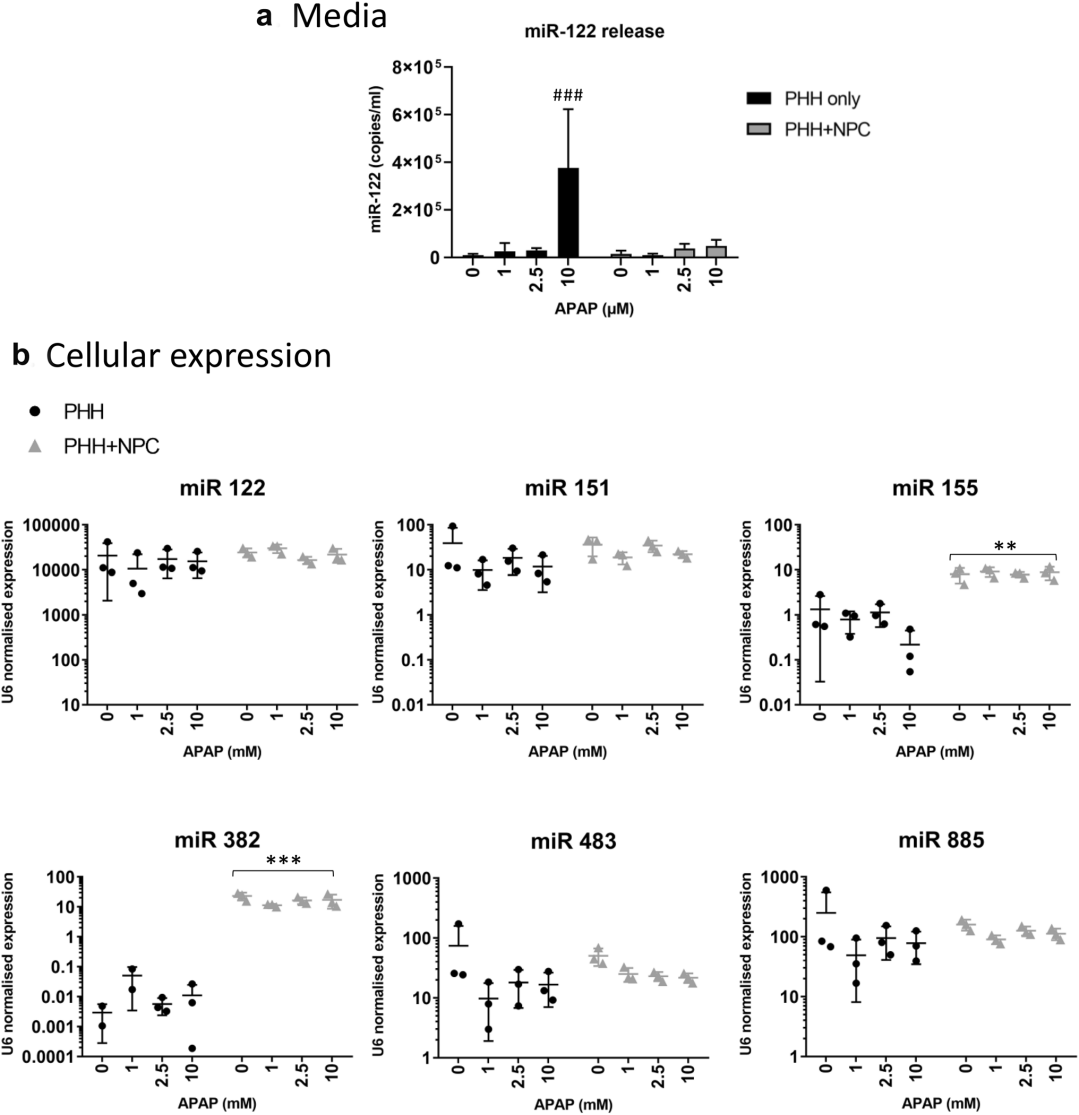
Changes in miRNA expression and release following APAP treatment are influenced by the presence of hepatic non-parenchymal cells. **a** Acetaminophen treatment results in release of miR-122 to the media, particularly in PHH only spheroids (black bars). Data represent the average of three biological replicates consisting media pooled from six spheroids each, following 72 h treatment. Of the six miRNAs investigated, only miR-122 was reliably detected in media samples. Ct values were interpolated from a standard curve generated from synthetic miR-122. **b** Cellular expression of miR-122, miR-151, miR-155, miR-382, miR-483 and miR-885 was assessed following 72 h APAP treatment. Six spheroids were pooled and lysed with Qiazol prior to qPCR with TaqMan Advanced reagents. Data represent the average of three biological replicates. U6 expression was used to normalise for RNA input. Expression was compared to untreated PHH spheroids and statistical significance was determined by Two-way ANOVA with corrections for multiple testing

miRNAs can serve as sensitive and specific biomarkers of drug-induced toxicity. Here, we investigated a panel of miRNAs, five of which have been associated with liver injury following APAP poisoning (miR-122, miR-151, miR-382, miR-483 and miR-885) (Vliegenthart et al. 2015) and one associated with inflammation (miR-155) (Bala et al. 2012). Plasma elevations in liver-enriched miRNAs are likely to reflect damage to hepatocytes and a leakage of cellular contents. Of the six miRNAs investigated, only miR-122 was reliably detected in media samples from APAP-treated spheroids (Fig. 4a). In support of the increased sensitivity observed in PHH only spheroids, the level of miR-122 release following APAP treatment was higher in PHH only spheroids compared to those containing additional NPCs, although miR-122 release was only induced at the highest APAP concentrations (Fig. 4a). Cellular levels of miR-122, however, were not affected, likely due to the very high levels present (Fig. 4b). Expression was equivalent in the two models.

Elevations in plasma miR-151 and miR-885 following APAP poisoning have also been reported previously (Vliegenthart et al. 2015). In our model, neither of these miRNAs were detected in media from a single spheroid, likely due to the limits of detection of the assay and much lower cellular expression compared to miR-122. For miR-151, APAP had no effect on cellular expression, and both models expressed this miRNA at equivalent levels (Fig. 4b). A slight reduction in miR-885 expression was observed following treatment of PHH only spheroids with APAP, however, this varied substantially between biological replicates (Fig. 4b).

Levels of miR-483 are reported to decrease in the plasma following APAP overdose (Vliegenthart et al. 2015). Here, APAP treatment appeared to cause a reduction in cellular expression of miR-483 in both models, this was of a greater magnitude in PHH only spheroids. This was not determined to be statistically significant however.

Both miR-155 and miR-382 were expressed at increased levels in spheroids containing NPCs (Fig. 4b). For miR-155, NPC-containing spheroids expressed approximately sixfold higher levels than PHH only spheroids at baseline. Treatment with the highest concentration of APAP (10 mM) resulted in a further reduction in miR-155 in PHH only spheroids. No such APAP effect was seen in spheroids containing NPCs. This may represent either a loss of miR-155 from damaged cells, or a downregulation in its expression. miR-382 was almost exclusively expressed in spheroids supplemented with NPCs. Under control conditions this equated to a > 7500-fold increase in expression compared to PHH only. No effect of APAP treatment was observed in either model.

### NPCs from multiple donors confer protection against APAP toxicity

**Fig. 5:**
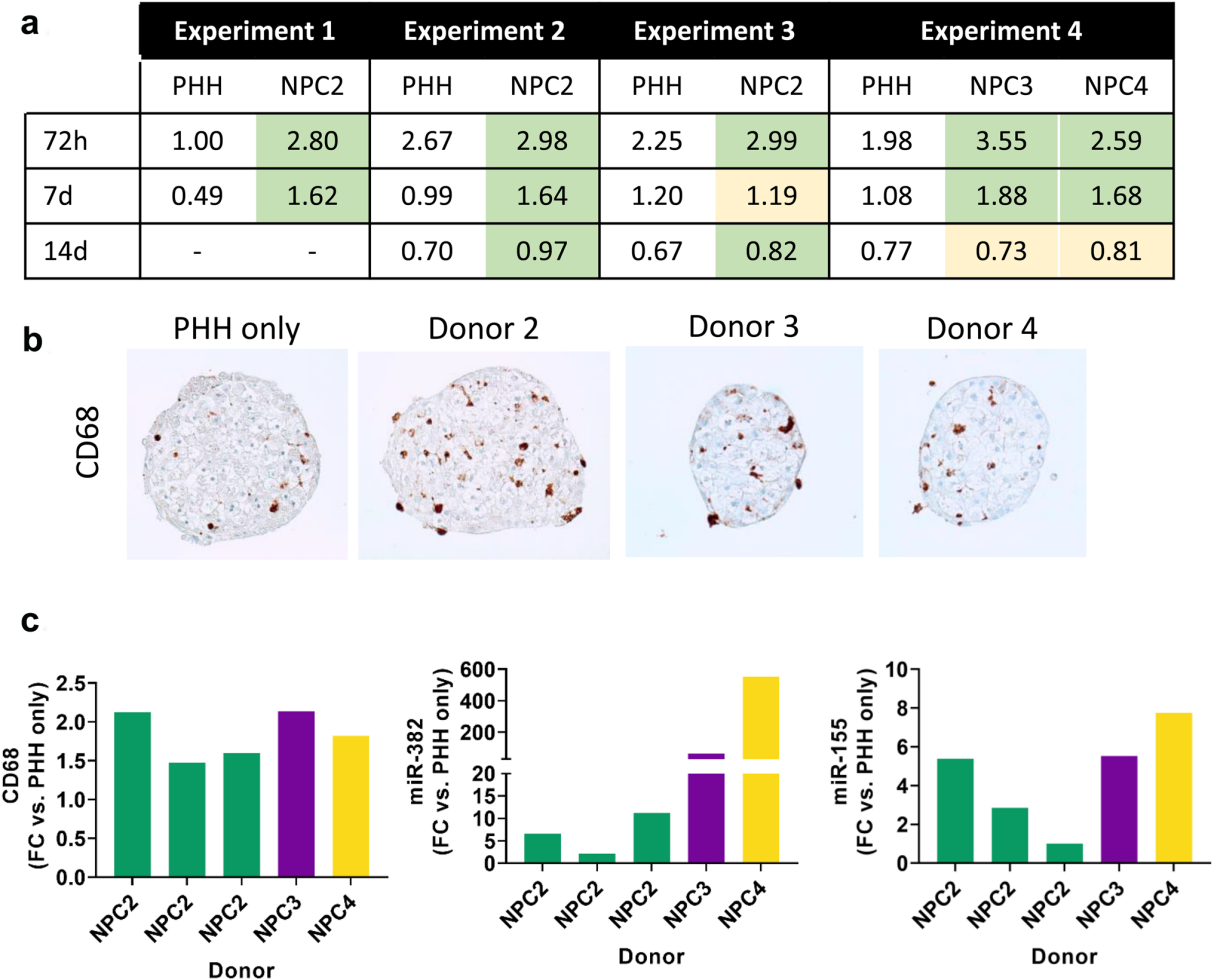
Non-parenchymal cells from multiple donors protect against APAP toxicity. **a** APAP cytotoxicity was compared through the calculation of EC_50_ values after 72 h, 7-day or 14-day treatment. NPC donors 3 and 4 were investigated in the same experiment and therefore are compared to the same PHH only spheroids. Green shading indicates a protective effect of NPCs. Yellow shading indicates equivalent toxicity in the two models. **b** The presence and distribution of Kupffer cells was assessed by IHC for the macrophage marker CD68. Some CD68 staining was observed in PHH only spheroids. This was increased with the introduction of supplementary NPCs. **c** The difference in mRNA expression of CD68 was quantitated by calculating the fold increase between NPC-containing cultures and PHH only spheroids in the same experiment with GAPDH used as a housekeeping gene. miR-382 and miR-155 were also present at higher levels in NPC-containing spheroids generated from all NPC donors

Donor-differences are important to consider when using human cells in vitro. We therefore obtained cryopreserved NPCs from three additional donors to see whether the protection against acetaminophen toxicity could be replicated. The same hepatocyte donor was used to remove any potential influence of differences in e.g. drug metabolizing enzyme expression. For all donors some degree of protection was conferred in the presence of supplementary NPCs (Fig. 5a; green shading) although the magnitude of this effect varied among donors and across timepoints. We attempted to explore the reasons behind these variations by comparing the expression of CD68 (Fig. 5b, c), miR-382, and miR-155 (Fig. 5c) between batches to understand whether it was possible to correlate the number and type of NPCs present to the protective effect seen. In all co-cultured spheroids increased levels of these markers were detected compared to hepatocyte only spheroids in the same experiment, however, there was no clear trend in terms of the level of marker expression and the response to APAP. Despite this, it may still be important to understand the relative numbers of eg. Kupffer cells, stellate cells, and LSECs for future studies incorporating NPCs—particularly when mixed preparations are used.

## Discussion

In vitro models incorporating human cells and/or tissue offer the possibility to investigate clinically important toxicity mechanisms at an early stage in drug development. The choice of culture system can, however, significantly impact cellular functionality and the expression of key phenotypic markers (Baker et al. 2018; Lauschke et al. 2016a, b; Lin and Khetani 2016). Culturing primary human hepatocytes as three-dimensional spheroids preserves many of the features which are rapidly lost in conventional 2D monolayers (Bell et al. 2016). However, despite this improved phenotype, most of the studies describing the use of spheroids for investigating DILI have predominantly focused on cytotoxicity endpoints rather than exploring how they can best be exploited to understand the underlying mechanisms.

Incorporating non-parenchymal cells into in vitro models may improve in vivo relevance, particularly where inflammation or innate immune responses are thought to be important (Godoy et al. 2013). In our present study, we demonstrated that introducing NPCs to PHH spheroids at the time of seeding did not have a detrimental effect on cellular viability and that the function of Kupffer cells and stellate cells could be retained. Titrating the ratio of PHH:NPC led us to selecting a ratio of 2:1 (1500 PHH and 750 NPC) based on the loss of response to LPS challenge when NPCs were included at lower numbers. This ratio has also been selected in other studies (Bell et al. 2016), although the actual physiological relevance of this ratio remains to be determined. Estimates of the number of different cell types present in the liver have suggested it is comprised of 60–65% hepatocytes, 15–20% endothelial cells, 10–15% Kupffer cells and 5–10% stellate cells (Dash et al. 2009; Bale et al. 2016), which is close to the ratio we have selected. In addition, actual ratios in vivo are likely to fluctuate depending on the current inflammatory status of the tissue. Another study focusing on screening DILI compounds for hepatotoxicity utilised spheroids containing 90% PHH and 10% NPC (Foster et al. 2019). In this study, the functionality of the NPCs was not assessed. The rationale for adding NPCs to PHHs may vary depending on the endpoints/mechanisms which are under investigation and in some instances, NPCs are merely added to improve spheroid formation. We selected mixed preparations of cryopreserved NPCs, assuming that the ratio of different NPC subtypes would be maintained in a similar proportion to the in vivo situation. However, the isolation processes selected may cause a bias towards certain cell types. Indeed, it is difficult to know whether any differences in NPC batches represent genuine differences in cellular composition between donors or rather occur as a result of the isolation process. Other models have included isolated preparations of Kupffer cells (Rose et al. 2016; Sunman et al. 2004) or stellate cells (Thomas et al. 2006), sometimes in combination with hepatic cell lines rather than primary hepatocytes. It is however unclear how stable a model including several different primary cell types from multiple different donors would be. For continued investigations into the role of non-parenchymal cells in hepatotoxicity we suggest that characterisation of cell types should be performed in all experiments. Ideally this should include phenotyping of the cultures using immunohistochemistry, to give a complete overview of the distribution of each cell type. For practical purposes, performing qPCR for a small panel of markers is likely to provide a reasonable measure of the variability of NPC composition between donors and experiments. Furthermore, although the same hepatocyte donor was used throughout the study, we found that the PHH fraction included contaminating NPCs, which in some preparations clearly impacted on the phenotype of the spheroids (Figs. 1, 5). This may contribute to the variability seen in some of the EC_50_ values between different experiments. The reproducibility of the model may be improved through using hepatic cell lines such as HepG2 or HepaRG (hepatocytes), LX-1 (stellate cells) or THP-1 (macrophages) (Prestigiacomo et al. 2017), at the expense of key cellular functionality only provided by primary cells.

One limitation of the spheroid model is that cells are mixed together in suspension and therefore it is not possible to control the exact localisation of each individual cell type. The proximity of each of the cell types and interaction between them may be important for some of their functionality eg. LSECs lining the sinusoids. For situations where a retained tissue architecture is vital, microphysiological systems (Foster et al. 2019) or bioprinting techniques (Ma et al. 2016; Nguyen et al. 2016) may provide ways to incorporate key anatomical features, such as the space of Disse. However, these systems in general have much lower throughput than 3D spheroids, which can be produced in 96- or 384-well plates. Using this relatively simple model, we have shown it is possible to maintain functional NPCs for a prolonged period as well as replicate toxicological effects that have been reported in vivo.

Acetaminophen toxicity has been widely studied as a model of drug-induced liver injury and is frequently used as an example of how metabolic activation and protein adduct formation can contribute to cell death. Non-parenchymal cells may be important in the toxicological response to APAP, and in particular the role of Kupffer cells has been widely discussed. Some groups have claimed that Kupffer cells exacerbate hepatic damage likely through the release of inflammatory mediators such as TNF-α (Ito et al. 2003; Laskin et al. 1995; Michael et al. 1999). This activation may occur directly or secondary to hepatocyte necrosis. However, these studies all used gadolinium chloride to inhibit the phagocytic activity of Kupffer cells, a compound which has been questioned due to its effects on reactive oxygen species formation (Jaeschke et al. 2012).

In another study, in which depletion of Kupffer cells was achieved with administration of liposomes loaded with clodronate, mice experienced more severe hepatotoxicity following APAP treatment, as indicated by histological changes and elevations in plasma ALT (Ju et al. 2002). The authors suggested that this was due to a reduction in the secretion of regulatory mediators such as IL-10. It is therefore likely that the balance between pro- and anti-inflammatory signals contributes to the severity of the liver damage caused. Additionally, activation of Kupffer cells may serve as an important signal in hepatocyte repair and regeneration (Jaeschke et al. 2012). Here, we have seen that mixed preparations of NPCs from multiple donors protect against APAP-induced hepatotoxicity. Although Kupffer cells are present in these preparations (as confirmed by CD68 staining), it cannot be ruled out that the other cell types may also contribute to the protective effect and there is some evidence that depletion of activated stellate cells with gliotoxin can result in more severe APAP liver injury (Shen et al. 2011).

Bioactivation to a protein-reactive imine, *N*-acetyl-*p*-benzoquinone (NAPQI), is a key step in the hepatic toxicity of APAP. NAPQI formation is associated with covalent binding to proteins, glutathione depletion and generation of reactive oxygen species which ultimately results in cellular necrosis. Microsomal incubations in both rat and human have attributed the formation of NAPQI to the cytochrome P450 enzymes: CYP2E1, CYP3A4, and CYP1A2 (Patten et al. 1993), although CYP2E1 appears to be the major enzyme involved in vivoin humans (Manyike et al. 2000). We quantified the amount of APAP-GSH formed in each of the cell models as a surrogate for NAPQI formation and found that levels of APAP-GSH were lower in spheroids which contained NPCs, suggesting either that less bioactivation occurs in this model or that APAP-GSH is more rapidly degraded in the presence of NPCs. At the same time mRNA expression of CYP1A2 (fourfold), CYP2E1 (threefold) and CYP3A4 (fourfold) was lower in NPC-containing spheroids, despite equivalent expression of the hepatocyte marker albumin. CYP expression was decreased further upon APAP treatment, likely either as a negative feedback mechanism or due to the cytotoxic effect of APAP. The reasons for the reduced expression of CYP enzymes in NPC-containing spheroids are however unclear. It is well established that cytokines can be involved in the downregulation of CYP450 enzymes (Klein et al. 2014) however, no IL-6 secretion was detected in the absence of LPS in either model.

MicroRNAs provide post-transcriptional regulation of gene expression through complimentary binding to the 3′-UTR of mRNA, resulting in either degradation of the transcript or repression of translation. The tissue specificity of some miRNAs has led to significant interest in their use as biomarkers of tissue injury, as circulating levels of a specific miRNA suggest leakage from damaged cells. Expression of miR-122 is almost entirely confined to the liver, where it makes up more than 70% of the total miRNA present (Lagos-Quintana et al. 2002) and in recent years it has emerged as a sensitive and specific marker of hepatic damage which can be monitored in the clinic, in animal studies and in vitro (Kia et al. 2015a, b; Lewis et al. 2011; Wang et al. 2009). Due to the small number of cells present in each spheroid it is perhaps unsurprising that the only miRNA reliably detected in cell culture media samples was miR-122. Previous in vitro studies in both 2D and 3D have indicated that miR-122 is at least as sensitive as ATP and LDH in reporting cytotoxicity (Foster et al. 2019; Kia et al. 2015a, b; Proctor et al. 2017), however currently it does not provide any additional mechanistic information. Where miR-122 may come into further importance is for example in the use of multi-organ in vitro systems where tissue-specific markers will be of significant value (Skardal et al. 2017).

When a random forest statistical model was applied to the miRNA profiling data obtained from the plasma of APAP poisoning patients, decreases in the plasma level of miR-382 best predicted patients that would experience severe liver injury (Vliegenthart et al. 2015). This is in direct contrast to miR-122, which was increased in the plasma, suggesting either that miR-382 is produced less or that it is degraded more quickly in patients with the most severe APAP liver injury. It could also be taken up into tissues. The presence of significantly higher levels of miR-382 in co-culture spheroids compared to PHH only spheroids is therefore an interesting finding and may point towards a potential role for miR-382 in the protective effect of NPCs. Increased expression of miR-382 in mouse liver has previously been observed following partial hepatectomy (Bei et al. 2015), with the authors highlighting that overexpression of miR-382 in hepatic cell lines resulted in increased proliferation, suggesting a possible role in liver regeneration. Limited studies currently exist which have looked at miR-382 in the liver, and even fewer that utilize primary human cells. CYP enzymes are not predicted targets of miR-382 (miRTarBase).

The study from Vliegenthart et al. (2015) did not identify miR-155 as being significantly dysregulated in APAP poisoning, however it was included in the current study due to its role in inflammation and immune cell development (Connell et al. 2012; O’Connell et al. 2007). It has also featured in a number of other studies looking at APAP toxicity, with miR-155^−/−^ mice experiencing more severe liver damage following APAP treatment (Yuan et al. 2016), likely due to an increase in expression of the pro-inflammatory cytokines TNF-a and IL-6. In general, the observation that NPCs contain different miRNAs to hepatocytes may be useful for understanding the mechanisms underlying drug toxicity or for identifying compounds that are toxic to a specific cell type. Unravelling the signalling pathways regulated by a specific miRNA will be challenging due to the fact that each miRNA has many mRNA targets.

To summarise, we have successfully introduced functional non-parenchymal cells into three-dimensional spheroids containing primary human hepatocytes and utilised this model to investigate APAP toxicity. The possibility to induce both inflammation and steatosis in NPC-containing spheroids highlights the potential value of co-cultures in investigating complex liver pathologies as well as confirming that expected signalling pathways are activated in the presence of model inducers. The protective effect of NPCs on APAP toxicity was observed via multiple endpoints and as such suggests that the interaction between multiple cell types can have important consequences for the response to hepatotoxins.

## Electronic supplementary material

Below is the link to the electronic supplementary material.

Supplementary file1 (DOCX 27 kb)

Supplementary file2 (PDF 153 kb)

## References

[CR1] Antoine DJ, Dear JW, Lewis PS, Platt V, Coyle J, Masson M, Park BK (2013). Mechanistic biomarkers provide early and sensitive detection of acetaminophen-induced acute liver injury at first presentation to hospital. Hepatology.

[CR2] Baker EJ, Beck NA, Berg EL, Clayton-jeter HD, Chandrasekera PC, Curley JL, Sullivan KM (2018). Advancing nonclinical innovation and safety in pharmaceutical testing. Drug Discov Today.

[CR3] Bala S, Petrasek J, Mundkur S, Catalano D, Levin I, Ward J, Szabo G (2012). Circulating MIcroRNAs in exosomes indicate hepatocyte injury and inflammation in alcoholic drug-induced, and inflammatory liver diseases. Hepatology.

[CR4] Bale SS, Geerts S, Jindal R, Yarmush ML (2016). Isolation and co-culture of rat parenchymal and non-parenchymal liver cells to evaluate cellular interactions and response. Sci Rep.

[CR5] Bei Y, Song Y, Wang F, Dimitrova-Shumkovska J, Xiang Y, Zhao Y, Yang C (2015). miR-382 targeting PTEN-Akt axis promotes liver regeneration. Oncotarget.

[CR6] Bell CC, Hendriks DFG, Moro SML, Ellis E, Walsh J, Renblom A, Fuhrmann A et al. (2016) Characterization of primary human hepatocyte spheroids as a model system for drug-induced liver injury, liver function and disease. Sci Rep 6:25187. https://www.nature.com/articles/srep25187 10.1038/srep25187 PMC4855186 27143246

[CR7] Connell RMO, Rao DS, Baltimore D (2012). microRNA regulation of inflammatory responses. Annu Rev Immunol.

[CR8] Dash A, Inman W, Hoffmaster K, Sevidal S, Kelly J, Obach RS, Tannenbaum SR (2009). Liver tissue engineering in the evaluation of drug safety. Expert Opin Drug Metab Toxicol.

[CR9] Foster AJ, Chouhan B, Regan SL, Rollison H, Amberntsson S, Andersson LC, Morgan P (2019). Integrated in vitro models for hepatic safety and metabolism: evaluation of a human Liver-Chip and liver spheroid. Arch Toxicol.

[CR10] Godoy P, Hewitt NJ, Albrecht U, Andersen ME, Ansari N, Bhattacharya S, Hengstler JG (2013). Recent advances in 2D and 3D in vitro systems using primary hepatocytes, alternative hepatocyte sources and non-parenchymal liver cells and their use in investigating mechanisms of hepatotoxicity, cell signaling and ADME. Arch Toxicol.

[CR11] Heslop JA, Rowe C, Walsh J, Sison-Young R, Jenkins R, Kamalian L, Kevin Park B (2016). Mechanistic evaluation of primary human hepatocyte culture using global proteomic analysis reveals a selective dedifferentiation profile. Arch Toxicol.

[CR12] Ito Y, Bethea NW, Abril ER, McCuskey RS (2003). Early hepatic microvascular injury in response to acetaminophen toxicity. Microcirculation.

[CR13] Jaeschke H, Williams CD, Ramachandran A, Bajt ML (2012). Acetaminophen hepatotoxicity and repair: the role of sterile inflammation and innate immunity. Liver Int.

[CR14] Ju C, Reilly TP, Bourdi M, Radonovich MF, Brady JN, George JW, Pohl LR (2002). Protective role of kupffer cells in acetaminophen-induced hepatic injury in mice. Chem Res Toxicol.

[CR15] Kia R, Kelly L, Sison-young RLC, Zhang F, Pridgeon CS, Heslop JA, Park BK (2015). MicroRNA-122: a novel hepatocyte-enriched in vitro marker of drug-induced cellular toxicity. Toxicol Sci.

[CR16] Kia R, Kelly L, Sison-Young RLC, Zhang F, Pridgeon CS, Heslop JA, Park BK (2015). MicroRNA-122: a novel hepatocyte-enriched in vitro marker of drug-induced cellular toxicity. Toxicol Sci.

[CR17] Klein M, Thomas M, Hofmann U, Seehofer D, Damm G, Zanger UM (2014). A systematic comparison of the impact of inflammatory signaling on ADME gene expression and activity in primary human hepatocytes and HepaRG cells. Drug Metab Dispos.

[CR18] Lagos-Quintana M, Rauhut R, Yalcin A, Meyer J, Lendeckel W, Tuschl T (2002) Identification of tissue-specific MicroRNAs from mouse. Curr Biol 12(9):735–739. https://www.sciencedirect.com/science/article/pii/S0960982202008096 10.1016/s0960-9822(02)00809-6 12007417

[CR19] Laskin DL, Gardner CR, Price VF, Jollow DJ (1995). Modulation of macrophage functioning abrogates the acute hepatotoxicity of acetaminophen. Hepatology.

[CR20] Lauschke VM, Hendriks DFG, Bell CC, Andersson TB, Ingelman-Sundberg M (2016). Novel 3D culture systems for studies of human liver function and assessments of the hepatotoxicity of drugs and drug candidates. Chem Res Toxicol.

[CR21] Lauschke VM, Vorrink SU, Moro SML, Rezayee F, Nordling Å, Hendriks DFG, Ingelman-Sundberg M (2016). Massive rearrangements of cellular MicroRNA signatures are key drivers of hepatocyte dedifferentiation. Hepatology.

[CR22] Lewis PJS, Dear J, Platt V, Simpson KJ, Craig DGN, Antoine DJ, Park BK (2011). Circulating microRNAs as potential markers of human drug-induced liver injury. Hepatology.

[CR23] Lin C, Khetani SR (2016). Advances in engineered liver models for investigating drug-induced liver injury. BioMed Res Int.

[CR24] Ma X, Qu X, Zhu W, Li Y-S, Yuan S, Zhang H, Chen S (2016). Deterministically patterned biomimetic human iPSC-derived hepatic model via rapid 3D bioprinting. Proc Natl Acad Sci.

[CR25] Manyike P, Kharasch E, Kalhorn T, Slattery J (2000). Contribution of CYP2E1 and CYP3A to acetaminophen reactive metabolite formation. Clin Pharmacol Ther.

[CR26] Martignoni M, Groothuis GMM, de Kanter R (2006). Species differences between mouse, rat, dog, monkey and human CYP-mediated drug metabolism, inhibition and induction. Expert Opin Drug Metab Toxicol.

[CR27] McGill MR, Lebofsky M, Norris HRK, Slawson MH, Bajt ML, Xie Y, Jaeschke H (2013). Plasma and liver acetaminophen-protein adduct levels in mice after acetaminophen treatment: dose-response, mechanisms, and clinical implications. Toxicol Appl Pharmacol.

[CR29] Messner S, Agarkova I, Moritz W, Kelm JM (2013). Multi-cell type human liver microtissues for hepatotoxicity testing. Archiv Toxicol.

[CR30] Michael SL, Pumford NR, Mayeux PR, Niesman MR, Hinson JA (1999). Pretreatment of mice with macrophage inactivators decreases acetaminophen hepatotoxicity and the formation of reactive oxygen and nitrogen species. Hepatology.

[CR31] Nguyen DG, Funk J, Robbins JB, Crogan-Grundy C, Presnell SC, Singer T, Roth AB (2016). Bioprinted 3D primary liver tissues allow assessment of organ-level response to clinical drug induced toxicity in vitro. PLoS One.

[CR32] O’Connell RM, Taganov KD, Boldin MP, Cheng G, Baltimore D (2007). MicroRNA-155 is induced during the macrophage inflammatory response. Proc Natl Acad Sci.

[CR33] Onakpoya IJ, Heneghan CJ, Aronson JK (2016). Post-marketing withdrawal of 462 medicinal products because of adverse drug reactions: a systematic review of the world literature. BMC Med.

[CR34] Park BK, Boobis A, Clarke S, Goldring CEP, Jones D, Kenna JG, Baillie TA (2011). Managing the challenge of chemically reactive metabolites in drug development. Nat Rev Drug Discov.

[CR35] Patten CJ, Thomas PE, Guy RL, Lee M, Gonzalez FJ, Guengerich FP, Yang CS (1993). Cytochrome P450 enzymes involved in acetaminophen activation by rat and human liver microsomes and their kinetics. Chem Res Toxicol.

[CR36] Prestigiacomo V, Weston A, Messner S, Lampart F, Suter-dick L (2017). Pro-fibrotic compounds induce stellate cell activation, ECM-remodelling and Nrf2 activation in a human 3D-multicellular model of liver fibrosis. PLoS One.

[CR37] Proctor WR, Foster AJ, Vogt J, Summers C, Middleton B, Pilling MA, Williams D (2017). Utility of spherical human liver microtissues for prediction of clinical drug-induced liver injury. Archiv Toxicol.

[CR38] Robinson MW, Harmon C, Farrelly CO (2016). Liver immunology and its role in inflammation and homeostasis. Cell Mol Immunol.

[CR39] Rose KA, Holman NS, Green AM, Andersen ME, Lecluyse EL (2016). Co-culture of hepatocytes and Kupffer cells as an in vitro model of inflammation and drug-induced hepatotoxicity. J Pharm Sci.

[CR40] Rowe C, Goldring CEP, Kitteringham NR, Jenkins RE, Lane BS, Sanderson C, Park BK (2010). Network analysis of primary hepatocyte dedifferentiation using a shotgun proteomics approach. J Proteome Res.

[CR41] Shen K, Chang W, Gao X, Wang H, Niu W, Song L, Qin X (2011). Depletion of activated hepatic stellate cell correlates with severe liver damage and abnormal liver regeneration in acetaminophen-induced liver injury. Acta Biochim Biophys Sin.

[CR42] Skardal A, Murphy SV, Devarasetty M, Mead I, Kang HW, Seol YJ, Atala A (2017). Multi-tissue interactions in an integrated three-tissue organ-on-a-chip platform. Sci Rep.

[CR43] Sunman JA, Hawke RL, LeCluyse EL, Kashuba ADM, CKanchagar, Seghezzi W, Evers R (2004). Establishment of a hepatocyte-Kupffer cell coculture model for assessment of proinflammatory cytokine effects on metabolizing enzymes and drug transporters. Drug Metab Dispos.

[CR44] Thomas RJ, Bhandari R, Barrett DA, Bennett AJ, Fry JR, Powe D, Shakesheff KM (2006). The effect of three-dimensional co-culture of hepatocytes and hepatic stellate cells on key hepatocyte functions in vitro. Cells Tissues Organs.

[CR45] Vliegenthart ADB, Shaffer JM, Clarke JI, Peeters LEJ, Caporali A (2015). Comprehensive microRNA profiling in acetaminophen toxicity identifies novel circulating biomarkers for human liver and kidney injury. Sci Rep.

[CR46] Wang K, Zhang S, Marzolf B, Troisch P, Brightman A, Hu Z, Galas DJ (2009). Circulating microRNAs, potential biomarkers for drug-induced liver injury. Proc Natl Acad Sci USA.

[CR47] Weaver RJ, Valentin J (2019). Today’s challenges to de-risk and predict drug safety in human “mind-the-gap”. Toxicol Sci.

[CR48] Yuan K, Zhang X, Lv L, Zhang J, Liang W, Wang P (2016). Fine-tuning the expression of microRNA-155 controls acetaminophen-induced liver inflammation. Int Immunopharmacol.

