# Supplementary material for: Functionality of primary hepatic non-parenchymal cells in a 3D spheroid model and contribution to acetaminophen hepatotoxicity
Source: Arch Toxicol. 2020 Feb 28;94(4):1251–63. doi: 10.1007/s00204-020-02682-w (PMC7225187; doi:10.1007/s00204-020-02682-w)
Supplement: Supplementary file 1 — Supplementary file1 (DOCX 27 kb) [file 204_2020_2682_MOESM1_ESM.docx]

Supplementary methods

**Cytokeratin 19 antibody:**

| **Antibody** | **Supplier** | **Host** | **Pretreatment** | **Dilution** |
| --- | --- | --- | --- | --- |
| CK19 | Dako | Mouse | stdCC1, pH8 | 1:100 |

**Determination of glutathione levels via LC-MS/MS:**

Spheroids were washed three times in DPBS and transferred to a new 96w plate in 30µl DPBS. An equal volume of monobromobimane (mBrB; 1.5mg/ml) was added together with 3 volumes of DPBS and samples were incubated for 40 min. Samples were quenched in acetonitrile containing 8nM of the internal standard 5,5-diethyl-1,3-diphenyl-2-iminobarbituric acid and 0.8% formic acid. Plates were centrifuged (4000g; 4 degrees; 20 min) and the resulting supernatant was diluted 1:3 in dd-H_2_O prior to analysis.

Quantification was achieved on a triple-quadrupole mass spectrometer (XevoTQ-XS; Waters, Milford, MA, USA) equipped with an Acquity ultraperformance liquid chromatography (UPLC) I-Class system (Waters Corporation, MA, USA). The MS system was equipped with an electrospray ionization source and settings were as follows: capillary voltage 0.5 kV; desolvation temperature 600°C; cone gas flow 150 l/h; nebulizer gas 7.0 bar; collision gas flow 0.15 ml/min. MSMS optimization was performed for all the analytes and conditions are specified in supplementary Table 2. Separation was performed using a Waters Atlantis UPLC® T3 column (50 mm×2.1 mm, 3 μm) fitted with a column heater set to 40°C. The mobile phase consisted of solvent A (0.1% formic acid in dd-H_2_O) and solvent B (0.1% formic acid in acetonitrile). The elution profile was: 0.2% B, 0.00 to 0.3 min; linear gradient to 95% B, 0.31 to 1.3 min; isocratic hold, 1.31 to 1.8 min; re-equilibration 5% B, 1.81 to 1.85 min. The flow rate was 1.0 ml/min. The injection volume was 2 µl.

| **Analyte** | **MRM (Parent→Daughter) m/z** | **Cone voltage (V)** | **Collision energy (V)** |
| --- | --- | --- | --- |
| 5,5-diethyl-1,3-diphenyl-2-iminobarbituric acid (IS) | 336.1→194.9 | 47 | 28 |
| GSH-mBrB | 498.2→369.1 | 10 | 20 |
