# Supplementary material for: Functionality of primary hepatic non-parenchymal cells in a 3D spheroid model and contribution to acetaminophen hepatotoxicity
Source: Arch Toxicol. 2020 Feb 28;94(4):1251–63. doi: 10.1007/s00204-020-02682-w (PMC7225187; doi:10.1007/s00204-020-02682-w)
Supplement: Supplementary file 2 — Supplementary file2 (PDF 153 kb) [file 204_2020_2682_MOESM2_ESM.pdf]

a.

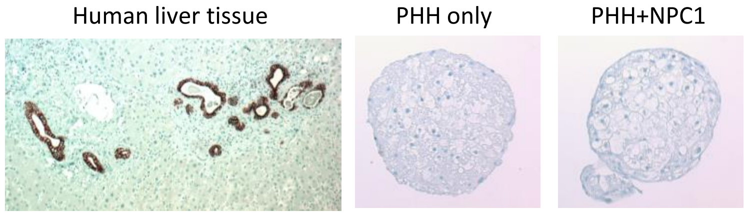

b.

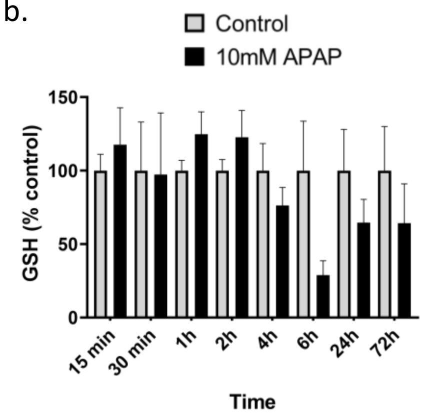

c.

	EC50 (mM)	
	-ABT	+ABT
24h	11.95	33.82
72h	3.94	4.19
7d	1.13	2.38
14d	1.06	1.54

Supplementary figure 1. Additional characterisation of primary human hepatocyte spheroids and response to acetaminophen treatment

a) Staining for cytokeratin-19 indicates that biliary epithelial cells are not present in the NPC preparations used in the current study.

b) Glutathione depletion following APAP treatment of PHH spheroids is detected after 4-6h. Total GSH was determined via LC-MS/MS following derivitaisation with monobromobimane. Each bar represents the average ± SD of 3-6 spheroids, and data is normalised to the untreated control (grey bars) at each timepoint.

c) Co-incubation with 1mM ABT (a non-selective CYP inhibitor) protects PHH spheroids from APAP cytotoxicity. Cellular ATP was measured from spheroids treated with 0-10mM APAP for the indicated timepoints, and the concentrations resulting in a 50% reduction in viability (EC50) were calculated.
